# Maturation-Dependent Changes in Volatile Aroma Profile and β-Glucosidase Activity in Kozan Misket Orange (*Citrus sinensis* L.)

**DOI:** 10.3390/metabo15110689

**Published:** 2025-10-24

**Authors:** Selin Yabacı Karaoğlan

**Affiliations:** Department of Food Engineering, Faculty of Engineering, Adana Alparslan Türkeş Science and Technology University, Adana 01250, Türkiye; syabaci@atu.edu.tr; Tel.: +90-4550000-2069

**Keywords:** β-glucosidase, HS-SPME/GC–MS, aroma-driven maturity, volatile compounds, oxygenated monoterpenes, regional citrus cultivar, sweet orange

## Abstract

Background/Objectives: Kozan Misket orange (*Citrus sinensis* L.) is a regional Turkish cultivar valued for its unique flavor, yet the mechanisms underlying its aroma development remain unclear. Volatile compounds are key contributors to citrus sensory quality, and β-glucosidase is involved in releasing glycosidically bound aroma precursors. However, no previous study has examined the interaction between enzyme activity and volatile production during maturation in this cultivar. This study aimed to characterize the dynamic changes in volatile composition and β-glucosidase activity across different maturation stages of Kozan Misket orange. Methods: Fruits were harvested at three maturity stages (green, green–yellow, yellow). Physicochemical traits (TSS, TA, TSS/TA), volatile profiles (HS-SPME/GC-MS), and specific β-glucosidase activity were analyzed. Volatile compounds were identified, quantified, and compared across stages. Results: A total of 47 volatile compounds were identified, with monoterpenes dominating at all stages. D-limonene was the most abundant compound, exceeding 86% of total volatiles. Total volatile content increased with maturation, particularly monoterpenes and sesquiterpenes, whereas oxygenated monoterpenes (e.g., linalool, 4-terpineol, α-terpineol) declined at full maturity. Specific β-glucosidase activity decreased markedly from 20.15 to 8.25 U mg^−1^ protein. This shift suggests that bound precursors contribute more to early-stage aroma release, while later-stage aroma accumulation may rely on metabolic conversions. Conclusions: This study provides the first integrated insight into aroma development in Kozan Misket orange, revealing a dual-phase mechanism linking volatile formation and β-glucosidase activity. These findings clarify cultivar-specific flavor development and offer guidance for harvest optimization and flavor management.

## 1. Introduction

**Figure 1:**
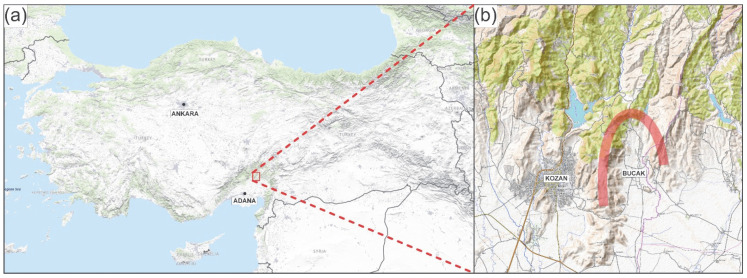
(**a**) Topographic map of Türkiye showing the location of Adana and Kozan within the eastern Mediterranean region. (**b**) Detailed topographic map of the Kozan district illustrating the Bucak area, which is situated at approximately 110 m above sea level in the transition zone between the Upper Plain of Adana and the foothills of the Taurus Mountains. Red line shows highlands surrounding the Bucak terroir. The area features semi-undulating terrain and a Mediterranean climate (hot, dry summers and mild, rainy winters), with an average annual precipitation of around 900 mm. Due to its sheltered position and fertile soils, this region provides a unique microclimate suitable for citrus cultivation. Panel (**a**) is based on the USGS Topographic Map (public domain; retrieved from https://basemap.nationalmap.gov/arcgis/rest/services/USGSTopo/MapServer, accessed on 20 November 2025). Panel (**b**) is based on OpenTopoMap (Geofabrik & OpenStreetMap contributors, licensed under CC-BY-SA 4.0), with proper attribution included.

The Kozan Misket orange (*Citrus sinensis*), a regional cultivar traditionally grown in the Bucak region of Kozan, Adana (Türkiye) (Figure 1), is distinguished by its characteristic flavor, high sugar content, and notably low acidity [1,2,3]. Unlike widely cultivated commercial orange types, this particular variety thrives solely under the specific microclimatic conditions of its native habitat—marked by moderate elevation, surrounding mountains, and optimal temperature gradients richness [4]. Despite its long-standing local significance—particularly in fresh consumption and artisanal products like orange wine—comprehensive scientific studies on its morphological, genetic, or biochemical attributes remain scarce. Turkey’s citrus industry is largely driven by commercial international varieties, leaving traditional types such as Kozan, Alanya, Finike, and Dörtyol relatively overlooked [2,3]. This is despite the country’s standing among the top ten global producers of oranges, where native varieties still represent a minor share in both domestic production and export [5].

Flavor plays a critical role in defining citrus fruit quality, shaped by a combination of sugars, acids, and volatile constituents—including terpenes, aldehydes, esters, and alcohols [6]. Volatile compounds, in particular, are highly responsive to maturation stages, environmental influences, and postharvest handling. In orange juice, D-limonene dominates the volatile profile, comprising 60–95% of the oil phase [7]. As fruits mature, sugar levels rise while acidity diminishes, thereby shifting the sugar-to-acid ratio—a crucial metric for gauging fruit maturation and flavor dynamics [8].

β-Glucosidase is an essential hydrolase that cleaves glycosidic bonds in various conjugated compounds. It plays a key role in releasing glycosidically bound aroma compounds (GBVs) such as monoterpene alcohols, as well as flavonoid glycosides, phenolic precursors, and other sugar-conjugated secondary metabolites. This enzymatic release enhances flavor expression and contributes to the formation of characteristic sensory profiles during fruit maturation [9]. These non-volatile, odorless conjugates—commonly referred to as glycosidically bound volatiles or “pro-aromas”—serve as latent aroma reservoirs that become active through enzymatic hydrolysis, primarily catalyzed by β-glucosidase [10]. This mechanism has been recognized as a central component of aroma development in a wide range of fruits, including orange, grape, strawberry, and mango, with substantial variation depending on cultivar, developmental stage, and tissue specificity [11]. In citrus fruits, peel tissues in particular have been shown to accumulate considerable amounts of GBVs that remain inactive until enzymatically released. For instance, Ren et al. [9] observed that β-glucosidase activity progressively increased during the ripening of Jincheng oranges and was strongly associated with the release of bound volatile compounds.

In addition to plant systems, the enzymatic release of aroma-active volatiles has been widely investigated in wine and juice matrices, where both microbial and endogenous β-glucosidases are known to enhance aroma intensity by hydrolyzing glycosidic precursors [3,12].

Despite these advances, the functional role of β-glucosidase in fruit maturation —particularly in citrus—and its link to the accumulation or modulation of free volatile aroma compounds remains underexplored. Moreover, no previous study has simultaneously examined β-glucosidase activity and volatile aroma profiles in Turkish citrus cultivars. This knowledge gap is particularly significant for Kozan Misket orange, a regional Turkish cultivar distinguished by its unique aromatic profile, yet it remains poorly characterized in the scientific literature. A more detailed understanding of how β-glucosidase influences aroma development during citrus maturation may shed light on key biochemical mechanisms underlying flavor biosynthesis and contribute to strategies aimed at improving juice quality and developing flavor-driven harvest and processing strategies. Therefore, this study investigates changes in β-glucosidase activity and volatile aroma composition across three maturation stages of Kozan Misket orange and explores the relationship between enzymatic activity and aroma compound formation in this traditional Turkish orange variety.

## 2. Materials and Methods

### 2.1. Plant Material

Kozan Misket orange (*Citrus sinensis*) fruits were collected at three defined maturation stages: green (28 September 2018), green-yellow (14 November 2018), and yellow (9 January 2019), from orchards situated in the Kozan district of Adana, Türkiye. Immediately following harvest, the samples were transported to the laboratory under refrigerated conditions and stored at −18 °C until analysis. The same freezing protocol was applied to all samples to maintain consistency across maturation stages. The sampling stages (green, green-yellow, yellow) were primarily based on peel color but were confirmed by TSS/acid ratio values (Table 1). Accordingly, the yellow stage corresponded to the fully mature (harvest) stage, whereas the green and green-yellow stages represented immature stages. Because no official maturity index exists specifically for Kozan Misket oranges, these stages were selected in consultation with an experienced agricultural engineer at the orchard to ensure horticultural relevance.

### 2.2. Fruit Quality Analysis

The pH of orange juice samples was measured using a Thermo Orion pH meter (Thermo Scientific, Waltham, MA, USA). Titratable acidity was assessed via titration with 0.1 N NaOH, employing phenolphthalein as the indicator, and expressed in grams of citric acid equivalents per 100 mL of juice. Soluble solids content (Brix (%)) was determined at 20 °C using a digital refractometer (Atago Co., Tokyo, Japan) after the samples were equilibrated to laboratory room temperature (20 °C), which is maintained year-round to ensure analytical consistency. All measurements were performed in triplicate following standard AOAC protocols [13].

### 2.3. Extraction and Identification of Volatile Compounds

Volatile compounds were extracted using headspace solid-phase microextraction (HS-SPME) and analyzed via gas chromatography–mass spectrometry (GC-MS). Juice samples were obtained by squeezing whole fruits with a standard laboratory juicer, and volatile compounds were analyzed from these juice samples. Volatile extraction was performed by mixing 5 mL of juice with 2 g of NaCl and 1 µL of 4-nonanol internal standard solution (1.108 µg/µL) in a 20 mL glass vial sealed with a PTFE/silicone septum [14]. The sample was shaken for 1 min, pre-incubated at 40 °C for 10 min, and then extracted for 30 min at 500 rpm. The adsorbed volatiles were desorbed in the GC injector at 250 °C for 5 min using splitless mode (split off for 0.8 min).

Analysis was carried out on an Agilent 7890B gas chromatograph coupled with an Agilent 6430 mass spectrometer (Agilent Technologies, Santa Clara, CA, USA). Separation was achieved using a DB-WAX capillary column (30 m × 0.25 mm i.d., 0.25 μm film thickness). The GC oven was programmed to hold at 40 °C for 4 min, then ramped to 90 °C at 2 °C/min, to 130 °C at 3 °C/min, and to 240 °C at 4 °C/min, followed by a final hold of 12 min. Injector and detector temperatures were maintained at 220 °C and 250 °C, respectively. Helium served as the carrier gas at a constant flow of 3 mL/min. Mass spectrometry was performed using electron impact ionization at 70 eV, with the ion source and quadrupole temperatures set at 250 °C and 120 °C, respectively. Spectra were recorded across a mass range of 29–350 *m*/*z*.

Volatile compounds were identified by comparing obtained spectra with the Wiley 7.0 and NIST libraries (John Wiley & Sons, Hoboken, NJ, USA; National Institute of Standards and Technology, Gaithersburg, MD, USA) and by calculating retention indices (Kovats index) against n-alkane standards. Where available, identification was confirmed using authentic reference compounds. All measurements were performed in triplicate. This method was adapted from Rambla et al. [14], which describes a validated HS-SPME/GC-MS protocol for citrus juice analysis.

### 2.4. Extraction, Partial Purification, and Activity Assay of β-Glucosidase

Fruit samples (300 g of peeled and deseeded pulp) were homogenized at low speed for 2 min using a commercial blender (Waring, Kendall County, TX, USA) in 400 mL of acetone pre-cooled to −18 °C by storage in a deep freezer and used immediately after removal, together with 3.33 g of polyethylene glycol. The mixture was vacuum filtered, and the remaining residue was re-extracted with 200 mL of cold acetone 2–3 times until a white acetone powder was obtained. The powder was then dried overnight at room temperature [15].

For partial purification, 5 g of the acetone powder was suspended in 400 mL of 0.1 M phosphate–citrate buffer (pH 6.8) containing 10 mM ascorbic acid, 4.0% (*w*/*v*) PVPP, 1.0% (*v*/*v*) Triton X-100, and 1 mM PMSF. The mixture was stirred at 4 °C for 4 h, followed by centrifugation at 10,000× *g* for 45 min at 4 °C with an Eppendorf 5810 R centrifuge (Eppendorf AG, Hamburg, Germany). The clear supernatant was treated with 85% ammonium sulfate saturation to induce precipitation [16].The precipitate was collected by centrifugation (10,000× *g*, 30 min, 4 °C), redissolved in a minimal volume of 0.01 M phosphate–citrate buffer (pH 6.8) sufficient to fully solubilize the precipitate, and dialyzed overnight at 4 °C against the same buffer [17,18].

Enzyme activity was determined spectrophotometrically by measuring the release of p-nitrophenol (pNP) from p-nitrophenyl-β-D-glucopyranoside at 400 nm using a Shimadzu UV-1700 spectrophotometer (Shimadzu Corp., Kyoto, Japan). For the assay, 0.1 mL of enzyme solution was mixed with 0.9 mL of 5 mM pNP-β-D-glucopyranoside in 0.1 M phosphate–citrate buffer (pH 6.8) and incubated at 50 °C for 15 min. The reaction was terminated by adding 1.0 mL of 1.0 M sodium carbonate. A blank sample containing only buffer was used as a control. Enzyme activity was calculated using a standard curve prepared with pNP concentrations ranging from 20 to 160 μM. All analyses were performed in triplicate. One unit (U) of β-glucosidase activity was defined as the amount of enzyme that releases 1 μL of pNP per minute under assay conditions [15].

### 2.5. Statistical Analysis

To assess the effect of maturation on individual volatile compounds, their concentrations at the three maturity stages (green, green-yellow, and yellow) were evaluated using one-way analysis of variance (ANOVA). When significant differences were detected (α = 0.05; *p* < 0.05), post hoc comparisons were performed using Tukey’s Honest Significant Difference (HSD) test. All statistical computations were carried out using Python 3.11 (Python Software Foundation, Wilmington, DE, USA) with the SciPy (Version 1.11) and Statsmodels libraries (Version 0.14) [19,20].

In addition, principal component analysis (PCA) was performed using XLSTAT (Version 2023.2; Addinsoft, Paris, France) to visualize multivariate patterns in volatile composition and to evaluate the contribution of individual aroma-active compounds to the separation of maturity stages. PCA was conducted using mean-centered data, and the first two principal components were used to generate score and loading plots.

## 3. Results

### 3.1. Fruit Quality Parameters During Maturation

To track maturation progression, pH, titratable acidity (TA, g citric acid equivalents per 100 mL of juice) and soluble-solids content (SSC, Brix (%) were measured at the green, green–yellow (immature stages), and yellow stages (the fully mature harvest stage) (Table 1).

As maturation advanced, pH and SSC gradually increased, while TA decreased (Table 1). This decrease in organic acids during citrus maturation is attributed to both metabolic catabolism and dilution due to increased juice content, as TA is expressed relative to juice volume (g citric acid/100 mL) rather than on a dry-weight basis [21,22].

### 3.2. Variation in Volatile Compounds During Maturation 

To examine the impact of maturation on aroma development, the volatile profile of Kozan Misket orange was analyzed at three maturity stages: green, green-yellow (immature stages), and yellow (the fully mature harvest stage) from fruit juice part. A total of 47 volatile compounds were identified across all stages, representing key chemical groups including monoterpenes, sesquiterpenes, aldehydes, aromatic hydrocarbons, and higher alcohols. Detailed information on each compound—such as linear retention indices (LRI), concentrations, ANOVA significance levels, and identification confidence (MS or MS+LRI)—are provided in Supplementary Table S1. The concentrations of volatile compounds grouped by chemical class across maturation stages are summarized in Table 2.

As seen in Table 2, maturation led to a marked increase in total volatile content, largely driven by elevated monoterpene levels. These compounds dominated the profile at every stage, rising from approximately 39,432 ± 415 µg/L in the green stage to over 50,878 ± 512 µg/L at mature stage. Table 3 provides a summary of key volatile compounds identified in Kozan Misket orange at different maturation stages.

### 3.3. β-Glucosidase Activity During Maturation

**Figure 2:**
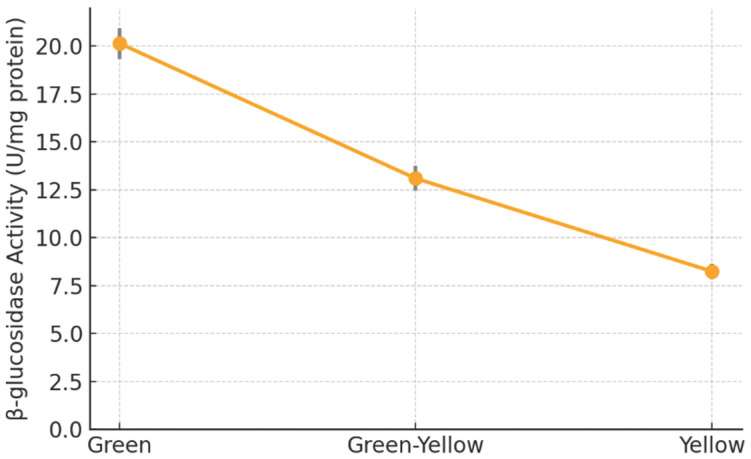
Stage-wise changes in β-glucosidase specific activity (U mg^−1^ protein) in Kozan Misket orange. Error bars represent the standard deviation of three biological replicates.

β-Glucosidases catalyze the hydrolysis of glycosidically bound aroma precursors, thereby shaping the volatile profile of maturing fruit [9]. In Kozan Misket orange, specific β-glucosidase activity declined steadily from 20.15 ± 0.80 U mg^−1^ protein at the green stage to 13.10 ± 0.65 U mg^−1^ at the green-yellow stage and 8.25 ± 0.40 U mg^−1^ at full maturity (Figure 2).

### 3.4. Multivariate Analysis (PCA)

**Figure 3:**
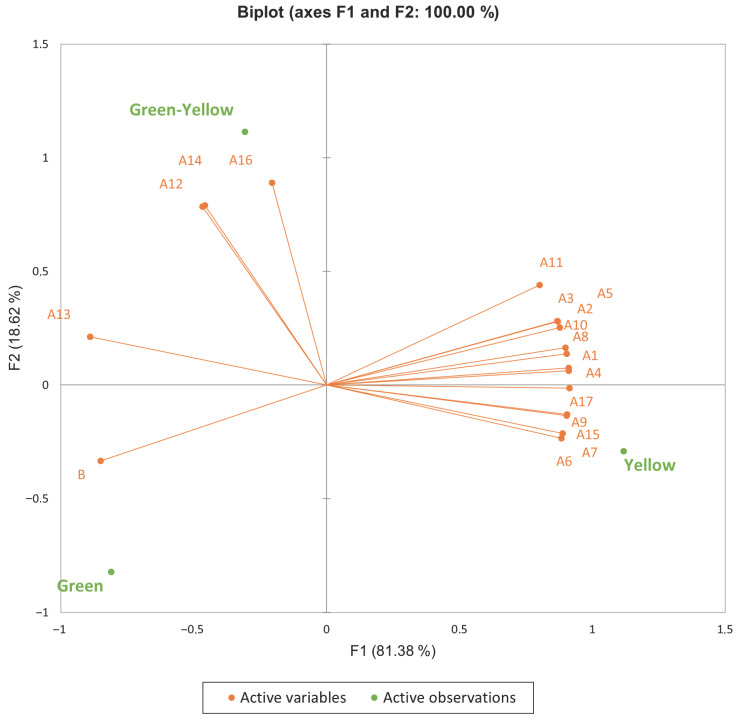
Principal component analysis (PCA) of volatile compounds (A1–A17) and β-glucosidase (B) during fruit maturation. Note: A1 = δ-3-Carene; A2 = D-Limonene; A3 = β-Phellandrene; A4 = γ-Terpinene; A5 = trans-Isolimonene; A6 = α-Terpinolene; A7 = trans-Geranylacetylene; A8 = β-Ocimene; A9 = 1,3,8-p-Menthatriene; A10 = Neo-allo-Ocimene; A11 = Limonene oxide; A12 = Linalool; A13 = 4-Terpineol; A14 = α-Terpineol; A15 = Valencene; A16 = Octanal; A17 = Decanal; B = β-Glucosidase.

To visualize the overall variation in volatile composition during maturation, a principal component analysis (PCA) was performed using the concentrations of major aroma-active compounds (Figure 3). The first two principal components (PC1 and PC2) explained 81.38% and 18.62% of the total variance, respectively, accounting for 100% of the variability in the dataset, which indicates an excellent representation of the data structure.

The score plot revealed a clear separation of samples according to ripening stage. Green samples were located on the negative side of PC1, yellow samples on the positive side, and green–yellow samples occupied an intermediate position. This progressive shift along PC1 demonstrates that maturation is the primary driver of changes in the volatile profile of Kozan Misket orange, which is consistent with the univariate results and the expected biochemical transitions during ripening.

## 4. Discussion

### 4.1. Fruit Quality Analysis of Kozan Misket Orange

The Brix-to-acid ratio (SSC/TA) showed a steady increase from 9.21 in the green stage to 15.58 at the yellow stage (corresponding to the fully mature harvest stage). While an SSC/TA ratio of 8–10.5 is generally accepted as the commercial-maturity range for sweet oranges [23,24], higher ratios (12–20) are often observed at full maturity depending on cultivar and growing conditions [24]. The ratio of 15.6 observed in Kozan Misket at the yellow stage indicates full maturity and favorable sweetness–acidity balance. According to the European Union quality parameters for citrus [24,25], juice content, total soluble solids (Brix), the soluble solids/acid ratio, and peel coloration are considered key indicators of citrus maturity and quality. The values obtained in this study are consistent with those expected for fully mature sweet oranges and support the selection of the yellow stage as the optimal harvest point.

### 4.2. Changes in Volatile Compounds During Maturation

D-Limonene remained the major volatile throughout, consistently accounting for ≥86% of the total volatile content, rising from 38,268 µg/L (green) to 49,013 µg/L (yellow), which is consistent with earlier reports identifying D-limonene as the predominant aroma compound in citrus species and showing its progressive increase during maturation [7]. Although its ortonasal odor threshold in orange juice is relatively high (~13,700 µg/L; Plotto et al. [26]), the concentrations observed here exceed this value by an order of magnitude, ensuring that D-limonene dominates the citrusy, licorice, “fresh-peel” aroma note and may also mask minor terpenes present below their thresholds [27,28].

Notably, whereas most monoterpenes increased with maturation, GBV-enriched monoterpene alcohols (e.g., linalool, α-terpineol, 4-terpineol) followed a different pattern: their levels peaked at the Green–Yellow stage and declined at full maturity. Similar stage-specific peaks have been reported in citrus and may reflect the progressive depletion of glycosylated precursors and/or shifts in terpene metabolism during later stages of maturation [6,29,30].

Same compounds—floral noted linalool (threshold in orange juice matrix [TOJ]: 113 µg/L; Plotto et al. [26]), minty-anise noted α-terpineol (TOJ: 25,900 µg/L, Plotto, et al. [26]; 0.35 µg/L in other matrices, Mai et al. [31]), woody-turpentine noted 4-terpineol (0.34 µg/L in other matrices; [31,32], and also lemon-resin notted δ-3-Carene (threshold, 0.77 µg/L in other matrices, Mai, et al. [31])—approached or exceeded their perception thresholds depending on the matrix and maturation stage [28,31]. For instance, linalool exceeded its TOJ threshold only at the green-yellow stage, while α-terpineol and 4-terpineol remained well below orange juice thresholds but may still contribute to aroma perception based on much lower values reported in other matrices. Although citrucy noted γ-terpinene was consistently below its TOJ threshold [26], its potential synergistic effects with other volatiles and matrix interactions may render it sensorially relevant. Therefore, the maturation-dependent behavior of these aroma-active volatiles has practical implications for determining optimal harvest timing and selecting fruit for high-flavor juice, essential oil extraction, or orange-wine production.

Additionally, compounds such as minty and herbaceous flavor noted β-phellandrene and piney α-terpinolene, these compounds were identified as aroma-active constituents in cold-pressed citrus oils. β-phellandrene showed a flavor dilution (FD) factor of 5 in GC-O/AEDA analysis, suggesting perceptual relevance, while terpinolene exhibited a relative flavor activity (RFA) of 5.3, indicating potential perceptual significance [33].

The increase in β-ocimene and neo-allo-ocimene concentrations during maturation contributes to the evolving aromatic profile of Kozan Misket orange. In our study, β-ocimene rose from 175.43 µg/L at the green stage to 238.12 µg/L at full maturity, while neo-allo-ocimene increased from 138.27 µg/L to 218.45 µg/L. The orthonasal odor threshold of β-ocimene has been reported as 34 µg/m^3^ in air [34], which is orders of magnitude lower than the concentrations measured in juice. While direct comparison is limited due to the different physical matrices (air vs. liquid), this disparity suggests a strong potential for β-ocimene to contribute to the sweet, minty, and juicy sensory notes [35] perceived in mature fruit. Although an odor threshold for neo-allo-ocimene could not be located in the literature, its co-accumulation with β-ocimene implies a potentially complementary role in shaping the late-stage citrusy-vegetal aroma [36].

In citrus, sesquiterpene biosynthesis mainly proceeds via the cytosolic mevalonate (MVA) pathway through farnesyl diphosphate (FPP), with possible minor contributions from plastidial MEP-derived precursors [37]. Total sesquiterpene concentration increased from 82 µg/L in green-yellow fruit to 730 µg/L in yellow fruit (*p* < 0.01; Tukey) and was below the detection limit at the green stage. Although valencene, was found at concentrations below the orthonasal odor thresholds (4756 µg/L) reported for orange juice [38], it may still contribute to the overall aroma perception due to synergistic interactions and matrix effects. Previous studies suggest that higher valencene levels are positively associated with fruit maturation and perceived aroma quality. Rather than being a dominant odorant per se, valencene may serve as a biochemical marker of maturity, indirectly indicating enhanced flavor complexity and oil quality in citrus fruits (e.g., oranges and mandarins) [35]. The delayed surge of valencene and α-selinene (undetectable at the green stage, modest at green-yellow, and abundant in yellow fruit) is consistent with the late transcriptional activation of sesquiterpene synthase genes in citrus. Valencene synthase (CsTPS1) is scarcely expressed in immature flavedo but rises sharply during color break and the fully mature stage, when increased cytosolic flux through the mevalonate (MVA) pathway supplies farnesyl diphosphate (FPP), the universal sesquiterpene precursor [39]. α-Selinene is a minor co-product of the same enzyme, and its accumulation therefore tracks that of valencene [39].

In plants, most volatile aldehydes originate either from the lipoxygenase (LOX), hydroperoxide lyase pathway of fatty-acid oxidation or from amino-acid catabolism (e.g., phenylalanine to phenylacetaldehyde) [40,41,42]. Although aldehydes are generally present in low concentrations, they are often considered disproportionately important in sweet-orange aroma due to their low odor thresholds and high odor-activity values (OAVs). In the current study, however, only octanal exceeded its orthonasal threshold at the green-yellow stage, while decanal remained below the threshold at all stages (threshold in OJ 204 µg/L [26]. Octanal, known for its minty, floral, and citrus-like aroma notes and an orthonasal threshold of approximately 233 µg/L in orange juice, increased from 180 µg/L at the green stage to 381 µg/L at the green-yellow stage, but then declined to near initial levels at full maturity [26].

Collectively, the rise in these medium-chain and aromatic aldehydes during late maturation intensifies the sweet, floral, and slightly resinous notes, balancing the dominant monoterpene core and contributing to the rounded sensory profile of Kozan Misket orange at the fully mature stage.

Aromatic hydrocarbons and several higher alcohols likewise increased during late maturation and may contribute complementary sweet, floral, resinous, or slightly woody nuances to the overall aroma [28,43].

From an application perspective, compared with other orange cultivars reported in the literature, Kozan Misket showed notably higher levels of valencene and δ-3-carene, which are key contributors to floral–resinous and complex aroma notes [44,45]. Previous studies have reported low or undetectable valencene levels in common commercial cultivars, whereas its marked increase in Kozan Misket suggests a cultivar-specific enrichment of sesquiterpene-derived aroma quality. In addition, the high abundance of other aroma-active compounds such as β-phellandrene, γ-terpinene, α-terpinolene, β-ocimene, linalool, 4-terpineol, and octanal further enhances the floral and fruity sensory characteristics of this cultivar, which is particularly important for the final quality of processed citrus-based products. The accumulation of key odor-impact compounds such as limonene and valencene, both of which play critical roles in citrus juice aroma and essential oil quality [35], indicates that fully mature Kozan Misket may offer superior sensory properties and potential suitability for flavor-focused products such as juice or orange-based fruit wine. Future olfactometric and sensory-guided analyses would help further elucidate the unique aroma-active components of this cultivar.

### 4.3. Discussion on β-Glucosidase Activity During Maturation 

The monotonic decrease suggests that the enzyme contributes mainly during early maturation, when the conversion of bound precursors is most active. Similar early-high/late-low patterns have been documented in strawberries [46], whereas some other cultivars—such as Jincheng oranges, which exhibit a gradual rise followed by a late decline [9] and Tarocco blood oranges, which peak at the late maturation stage [47]—show a later maximum. These contrasting trends highlight the influence of cultivar-specific physiological regulation on β-glucosidase activity.

Similar stage-dependent declines have been reported for other fruit glycosidases, including α-mannosidase and β-galactosidase in grapefruit [48], suggesting a broader regulatory pattern. Nonetheless, direct studies linking β-glucosidase to aroma formation remain scarce, and our data support its primary role during early maturation when precursor hydrolysis is most active.

The observed stage-dependent decrease in β-glucosidase activity, coupled with the transient peak of monoterpene alcohols (linalool, α-terpineol, 4-terpineol) at the intermediate stage (Green–Yellow), indicates that enzymatic hydrolysis of glycosidically bound precursors occurs mainly during early to mid-maturation (Stage 1 → Stage 2) as similarly reported in strawberries [46]. In contrast, the decline in both β-glucosidase activity and monoterpene alcohols from Stage 2 to Stage 3 suggests that later changes in the volatile profile are likely driven by de novo biosynthesis, storage/release dynamics (reduced contributions from GBV pools), or downstream metabolic conversion rather than continued glycoside cleavage [9].

By comparing the trends of volatiles and enzyme activity across the same maturation stages (Stage 1–Stage 2–Stage 3), a complementary pattern emerges: β-glucosidase plays a key role in shaping the volatile profile during the initial maturation phase, while other metabolic processes dominate later stages. This complementary pattern provides a biochemical basis for examining both free volatiles and β-glucosidase activity within the same study.

This dual-phase behavior complements the observed maturation-dependent shifts in free terpenes, particularly the early peak of glycosidically derived monoterpene alcohols (e.g., linalool, α-terpineol) and the late accumulation of de novo synthesized sesquiterpenes (e.g., valencene), linking enzymatic activity directly to aroma development. Similar trends have been observed in other fruit species [9,46]. Although β-glucosidase is not employed as a routine maturity index, its stage-dependent decline, together with the compositional shifts, highlights its potential value as an aroma-related maturity marker for flavor-driven cultivars like Kozan Misket, as noted by Gupta et al. [23].

### 4.4. Discussion of Multivariate Analysis (PCA)

Since PCA revealed distinct volatile profiles across ripening stages, the loading plot was examined to elucidate which aroma-active compounds and enzyme activity patterns drove these stage-specific differences. The loading plot further clarified which compounds characterized each stage. Compounds such as D-limonene (A2), β-phellandrene (A3), γ-terpinene (A4), trans-isolimonene (A5), neo-allo-ocimene (A10), limonene oxide (A11), decanal (A17), and valencene (A15) loaded strongly in the positive direction of PC1 and were associated with the fully ripe (yellow) stage, reflecting the accumulation of citrus-like, sweet, and complex aroma notes. In contrast, 4-terpineol (A13) and β-glucosidase (B) loaded on the negative side of PC1 together with the green stage, indicating lower aroma complexity and enzyme activity in immature fruit.

Interestingly, linalool (A12), α-terpineol (A14), octanal (A16), and 1,3,8-p-menthatriene (A9) loaded more strongly on PC2, suggesting that these compounds were more prominent in the green–yellow (mid-ripening) stage, possibly reflecting transitional metabolic activity and the early onset of aroma diversification.

Overall, the PCA results demonstrate that each maturity stage exhibits a distinct volatile fingerprint, and specific aroma-active compounds drive the separation of stages. These findings support the hypothesis that Kozan Misket develops a characteristic aroma profile during maturation and provide multivariate confirmation of the cultivar’s unique volatile composition.

## 5. Conclusions

This study provides the first integrated characterization of volatile aroma development and β-glucosidase activity in Kozan Misket orange, an underexplored Turkish sweet orange cultivar valued for its low acidity and high sugar content. A total of 47 volatile compounds were identified, with D-limonene as the dominant constituent and overall volatile release intensifying with maturation. Maturation also led to qualitative shifts across chemical classes, particularly enhanced sesquiterpenes and transient increases in oxygenated monoterpenes. β-Glucosidase activity decreased by ~60% from green to yellow stages, indicating that precursor hydrolysis is more relevant in early development, whereas later aroma accumulation is likely driven by biosynthetic and metabolic pathways. In addition, PCA confirmed that each maturity stage exhibited a distinct volatile fingerprint driven by specific aroma-active compounds, providing multivariate support for the cultivar-specific nature of flavor development. These findings enhance understanding of cultivar-specific aroma formation and highlight maturity as a key determinant of flavor quality. Overall, the results not only expand citrus flavor knowledge but also provide practical guidance for optimizing harvest timing and valorizing regional germplasm. Future studies incorporating sensory evaluation and more comprehensive biochemical analyses will further illuminate the unique attributes of this little-studied cultivar. Given its distinctive flavor profile, Kozan Misket may hold strong potential not only for fresh consumption but also for the production of high-quality fruit wines, which could increase its added value and enhance regional valorization.

## Figures and Tables

**Table 1:** Fruit Quality Analysis (mean ± standard deviation) of Kozan Misket oranges at different maturation stages. Green and green-yellow correspond to immature stages, while yellow represents the fully mature (harvest) stage.

Maturation Stage	pH (Mean ± SD)	Titratable Acidity (g Citric Acid/100 mL ± SD)	Brix (%) (Mean ± SD)	SSC/TA Ratio
Green	3.22 ± 0.01 ^c^	1.18 ± 0.01 ^a^	10.87 ± 0.10 ^b^	9.21 ^c^
Green-Yellow	3.45 ± 0.01 ^b^	0.96 ± 0.01 ^b^	10.33 ± 0.15 ^c^	10.76 ^b^
Yellow	3.50 ± 0.01 ^a^	0.77 ± 0.03 ^c^	12.00 ± 0.10 ^a^	15.58 ^a^

Green and green-yellow correspond to immature stages, while yellow represents the fully mature (harvest) stage. Different lowercase letters indicate significant differences among maturity stages at α = 0.05 (ANOVA, Tukey’s HSD).

**Table 2:** Total volatile concentrations (µg/L) by chemical class across three maturation stages of Kozan Misket orange.

Chemical Class	Green (µg/L)	Green-Yellow (µg/L)	Yellow (µg/L)
Monoterpenes	39,431.51 ± 2962.02 ^a^	45,215.47 ± 2255.85 ^ab^	50,878.07 ± 3421.31 ^b^
Sesquiterpenes	0.00 ± 0.00 ^a^	81.54 ± 15.13 ^b^	778.20 ± 21.04 ^c^
Aromatic Hydrocarbons	2995.88 ± 263.66 ^a^	3302.12 ± 195.05 ^ab^	4709.54 ± 788.29 ^b^
Aldehydes	246.01 ± 45.16 ^a^	473.24 ± 46.36 ^b^	323.38 ± 43.96 ^a^
Higher Alcohols	295.75 ± 18.60 ^b^	540.67 ± 8.11 ^c^	193.74 ± 10.28 ^a^
Total Volatiles	42,969.14 ± 3289.44 ^a^	49,613.04 ± 2520.50 ^ab^	56,882.94 ± 4284.88 ^b^

Green and green-yellow correspond to immature stages, while yellow represents the fully mature (harvest) stage. Different lowercase letters indicate significant differences among maturity stages at α = 0.05 (ANOVA, Tukey’s HSD). Groups sharing at least one letter (e.g., “ab”) are not significantly different.

**Table 3:** Concentrations (µg/L) of key volatile compounds in Kozan Misket orange at three maturation stages.

LRI	Compound	Green (µg/L)	Green-Yellow (µg/L)	Yellow (µg/L)	Significance
1171	δ-3-Carene	134.97 ± 11.82 ^a^	184.35 ± 28.81 ^a^	286.98 ± 32.59 ^b^	**
1197	D-Limonene	38,268.14 ± 2858.08 ^a^	43,747.03 ± 2143.37 ^a^	49,012.73 ± 3048.85 ^b^	**
1207	Β-Phellandrene	156.29 ± 14.36 ^a^	173.72 ± 14.97 ^a^	188.61 ± 24.86 ^a^	ns
1250	γ-Terpinene	32.89 ± 13.66 ^a^	47.64 ± 17.32 ^a^	76.58 ± 23.64 ^a^	ns
1268	Trans-isolimonene	37.48 ± 2.44 ^a^	50.34 ± 1.99 ^b^	61.44 ± 4.44 ^c^	***
1274	α-Terpinolene	175.11 ± 30.90 ^a^	174.39 ± 20.73 ^a^	294.04 ± 83.98 ^a^	ns
1277	Trans-geranylacetylene	17.40 ± 1.64 ^a^	20.25 ± 1.75 ^a^	140.97 ± 22.48 ^b^	***
1279	β-Ocimene	175.15 ± 9.74 ^a^	200.30 ± 8.83 ^b^	238.27 ± 34.85 ^b^	*
1286	1,3,8-p-Menthatriene	21.01 ± 1.40 ^a^	31.81 ± 1.40 ^a^	108.19 ± 102.53 ^a^	ns
1296	Neo-Allo-Ocimene	137.86 ± 10.06 ^a^	171.80 ± 0.57 ^b^	217.89 ± 6.60 ^c^	***
1407	Limonene oxide	22.13 ± 0.00 ^a^	46.12 ± 0.74 ^b^	56.10 ± 3.73 ^c^	***
1545	Linalool	74.51 ± 2.39 ^b^	141.08 ± 7.42 ^c^	49.44 ± 2.37 ^a^	***
1609	4-Terpineol	118.87 ± 2.36 ^b^	117.26 ± 3.65 ^b^	53.23 ± 14.78 ^a^	***
1690	α-terpineol	59.71 ± 3.16 ^b^	109.38 ± 4.30 ^c^	41.98 ± 5.04 ^a^	***
1707	Valencene	0.00 ± 0.00 ^a^	81.54 ± 15.13 ^b^	692.43 ± 7.83 ^c^	***
1284	Octanal	179.82 ± 37.71 ^a^	381.09 ± 42.76 ^b^	189.41 ± 21.34 ^a^	***
1495	Decanal	66.19 ± 7.45 ^a^	67.90 ± 1.56 ^a^	73.09 ± 14.17 ^a^	ns

Green and green-yellow correspond to immature stages, while yellow represents the fully mature (harvest) stage. Values represent mean ± SD (*n* = 3). Different lowercase superscript letters within a row indicate significant differences among maturation stages (one-way ANOVA, Tukey’s HSD, α = 0.05). Significance levels: ns = not significant (*p* > 0.05); * *p* ≤ 0.05; ** *p* ≤ 0.01; *** *p* ≤ 0.001. Compounds were identified using mass spectral data and linear retention indices (LRI) from NIST and Wiley libraries; authentic standards were used when available.

## Data Availability

The data presented in this study are available in the article and its Supplementary Materials. Additional raw data supporting the findings are available from the corresponding author upon reasonable request.

## Supplementary Materials

The following supporting information can be downloaded at: https://www.mdpi.com/article/10.3390/metabo15110689/s1, Table S1: Concentrations (µg/L) of volatile compounds at three maturation stages of Kozan Misket orange.

## Abbreviations

The following abbreviations are used in this manuscript:

ATÜ-BAP	Adana Alparslan Türkeş Science and Technology University—Scientific Research Projects Unit
AOAC	Association of Official Analytical Chemists
ANOVA	Analysis of Variance
DB-WAX	Polyethylene Glycol-Based Capillary GC Column (Brand: DB-Wax)
FPP	Farnesyl Diphosphate
GBVs	Glycosidically Bound Volatiles
GC-MS	Gas Chromatography–Mass Spectrometry
HS-SPME	Headspace Solid-Phase Microextraction
LRI	Linear Retention Index
MVA	Mevalonate Pathway
OAV	Odor Activity Value
OJ	Orange Juice
pNP	p-Nitrophenol
PMSF	Phenylmethylsulfonyl Fluoride
PTFE	Polytetrafluoroethylene
PVPP	Polyvinylpolypyrrolidone
SD	Standard Deviation
SSC	Soluble Solids Content
TA	Titratable Acidity
TOJ	Threshold in Orange Juice matrix
U	Enzyme Unit
